# Atypical Macropinocytosis Contributes to Malignant Progression: A Review of Recent Evidence in Endometrioid Endometrial Cancer Cells

**DOI:** 10.3390/cancers14205056

**Published:** 2022-10-15

**Authors:** Takayuki Kohno, Takashi Kojima

**Affiliations:** Department of Cell Science, Research Institute for Frontier Medicine, Sapporo Medical University School of Medicine, Sapporo 060-8556, Japan

**Keywords:** endometrioid-endometrial carcinoma, endometriosis, macropinocytosis, LSR, tight junctions, dormancy

## Abstract

**Simple Summary:**

A novel type of macropinocytosis has been identified as a trigger for the malignant progression of endometrial cancer. Transiently reducing epithelial barrier homeostasis leads to macropinocytosis by splitting between adjacent cells in endometrioid endometrial cancer. Macropinocytosis causes morphological changes in well-differentiated to poorly differentiated cancer cells. Inhibition of macropinocytosis promotes a persistent dormant state in the intrinsic KRAS-mutated cancer cell line Sawano. This review focuses on the mechanisms of atypical macropinocytosis and its effects on cellular function, and it describes the physiological processes involved in inducing resting conditions in endometrioid endometrial cancer cells.

**Abstract:**

Macropinocytosis is an essential mechanism for the non-specific uptake of extracellular fluids and solutes. In recent years, additional functions have been identified in macropinocytosis, such as the intracellular introduction pathway of drugs, bacterial and viral infection pathways, and nutritional supplement pathway of cancer cells. However, little is known about the changes in cell function after macropinocytosis. Recently, it has been reported that macropinocytosis is essential for endometrial cancer cells to initiate malignant progression in a dormant state. Macropinocytosis is formed by a temporary split of adjacent bicellular junctions of epithelial sheets, rather than from the apical surface or basal membrane, as a result of the transient reduction of tight junction homeostasis. This novel type of macropinocytosis has been suggested to be associated with the malignant pathology of endometriosis and endometrioid endometrial carcinoma. This review outlines the induction of malignant progression of endometrial cancer cells by macropinocytosis based on a new mechanism and the potential preventive mechanism of its malignant progression.

## 1. Introduction

Macropinocytosis has long been known to be one of the mechanisms involved in the uptake of extracellular components. This phenomenon was discovered in macrophages in 1931 and is a widely confirmed biological phenomenon from amoebas and slime molds to mammalian cells [1]. Endocytosis is the mechanism by which cells incorporate extracellular factors. Endocytosis is an essential mechanism for the uptake of the extracellular components required for housekeeping tasks. According to the typical definition, they are classified into two types: phagocytosis, which involves the uptake of solid substances, and pinocytosis, which involves the uptake of fluid components [2]. Macropinocytosis is a process that nonspecifically incorporates extracellular components on a large scale. Macropinocytosis is a physiologically important function of immune cells, endothelial cells, and epithelial cells. In cancer cells, high macropinocytosis activity is used as a supply pathway of amino acids required for cell proliferation [3]. A recent study identified a new role for macropinocytosis, inducing the initiation of malignant progression of endometrial cancer cells [4]. Macropinocytosis is attributed to the disruption of epithelial cell homeostasis. Evidence that macropinocytosis contributes to the malignant process may point toward new targets for the prevention of cancer.

## 2. Uptake Mechanisms of Extracellular Components

**Figure 1:**
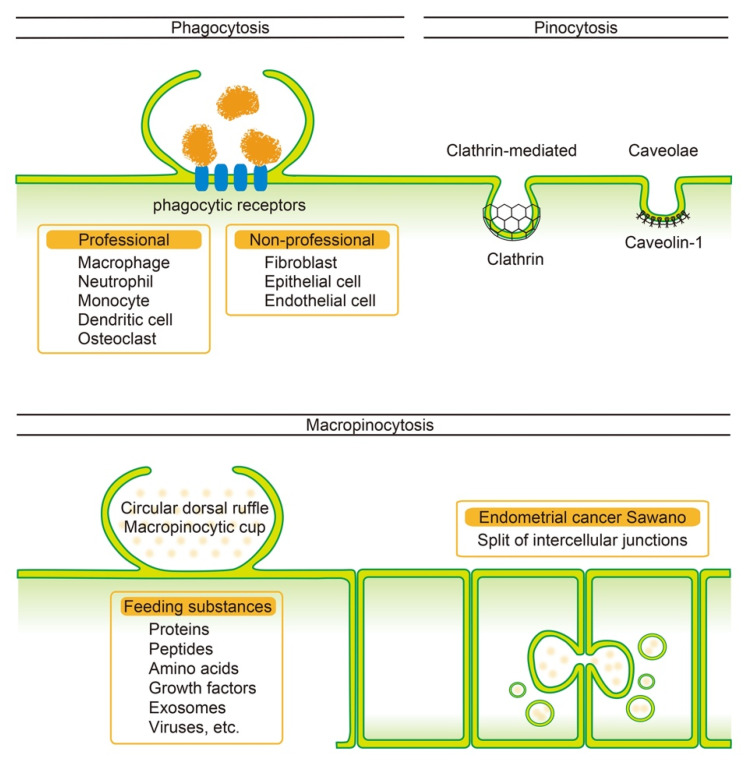
Overview of the endocytic pathways. Phagocytosis incorporates extracellular components through phagocytic receptors. Cells that phagocytose with high efficiency are termed professional cells, whereas those with low efficiency are termed non-professional cells. Pinocytosis nonspecifically incorporates soluble components, such as proteins and growth factors. Small vesicles are formed through clathrin- and caveolae-mediated pathways during pinocytosis. The pathway of extensive incorporation, with membrane protrusion from the cell surface, is termed macropinocytosis. The endometrial cancer cell Sawano exhibits macropinocytosis caused by a transient split of intercellular spaces.

Most phagocytic cells actively incorporate the target factors (Figure 1) [5]. Professional phagocytes, such as macrophages, neutrophils, and dendritic cells, and non-professional phagocytes, such as fibroblasts and epithelial cells, incorporate targets, which include solid components containing apoptotic cells, through receptor-mediated endocytosis [6]. Professional phagocytes efficiently engulf extracellular components through complement-mediated opsonization. Phagosome formation involves membrane ruffling via PI3K-mediated actin reorganization [7]. The coordinated reconstruction of cell membranes is also required [8]. *Mycobacterium tuberculosis* uses phagocytosis as a route of entry into the cell, but its mechanism is different from that of foreign body removal to prevent its own degradation [9]. Some cancer cells express CD47 [10]. The interaction of CD47 with the SIRPα of macrophages renders cancer cells resistant to phagocytosis, thereby promoting tumor growth and metastasis [11]. Endometrial cancer also expresses CD47, and it has been pointed out that its inhibition may be a therapeutic target [12].

Pinocytosis can be classified according to the size of vesicles formed [13]. Small vesicle-mediated pinocytosis is further classified into the following three types: clathrin-mediated, caveolae pathways and those mediated by neither. In the clathrin-mediated pathway, clathrin-based triskelion vesicles facilitate intracellular transport [14]. Caveolae form vesicles associated with caveolin-1, which are smaller than clathrin-coated vesicles [15]. These small vesicles originate from concavity of the cell membrane [16]. Pinocytosis, accompanied by the formation of large vesicles, is called macropinocytosis. Macropinocytosis does not require coat proteins such as clathrin for uptake. In addition, it is believed that there is no communication mechanism or interaction between the incorporated target and the cell [17]. In macropinocytosis, extracellular factors are incorporated into membrane protrusions via a pathway that involves extensive actin rearrangement [18] and membrane ruffling [19]. A mathematical model has been developed to explain the mechanism of formation of a large macropinocytic cup during macropinocytosis [20].

The extracellular uptake mechanism is used as a route for viral infection [21]. Phagocytosis allows herpes simplex viruses (HSV-1) and acanthamoeba polyphaga mimivirus (AMPV) to enter cells, whereas in clathrin-mediated endocytosis, human immunodeficiency virus type 1 (HIV-1) and influenza A virus (IAV) enter cells, while simian virus (SV40) and BK virus enter via caveolae. Adenovirus 3 (Ad3), coxsackievirus B (CVB), and respiratory syncytial virus (RSV) enter cells through macropinocytosis [21,22,23].

## 3. A Universal Role of Macropinocytosis

Among immune system cells, macrophages and dendritic cells constantly perform macropinocytosis to eliminate foreign substances and present antigens [24]. B cells, CD4^+^ T cells, and CD8^+^ T cells utilize macropinocytosis for their physiological functions [25,26].

In fibroblasts, growth factors, such as PDGF and EGF, stimulate Ras and Src via activation of RTKs, leading to macropinocytosis [27,28]. Circular dorsal ruffles (CDRs) occur on the cell surface and are involved in the formation of macropinocytic cups [29]. The PI3K-Akt-TSC1-Rheb-mTORC1 pathway is associated with growth factor-dependent macropinosome formation [30]. The macropinosome fuses with the lysosome, and its contents are absorbed into the cell [3]. mTORC1/2 complexes regulate the recycling of essential biomolecules in lysosomes through interactions with the nutrient source sensor TSC1/2 [31,32].

In endothelial cells, macropinocytosis is a pathway for growth factor receptor internalization in a growth factor-dependent manner. Cell surface VEGFR2 is incorporated via macropinocytosis by Cdc42 activation upon VEGF stimulation [33]. Cell surface FGFR1 is incorporated into Rab5-positive endosomes by macropinocytosis via RhoG activation upon FGF2 stimulation [34]. Macropinocytosis is required for the activation of intracellular signaling pathways that are dependent on growth factor stimulation.

Since collective epithelial cells have established cell polarity, they incorporate extracellular components from the apical and basal membranes. In polar MDCK cells, uptake occurs via clathrin-mediated and clathrin-independent pathways on the apical surface, whereas caveolae-mediated uptake occurs on the basal membrane [13]. Two types of plasma membrane ruffles precede macropinocytosis: circular dorsal ruffles (CDRs) arising from the apical cell surface and planar ruffles arising from the cell periphery [3]. These processes involve the activation of the RhoG-Rac pathway and reorganization of actin filaments [35]. Recently, it was reported that macropinocytosis is necessary for breaking out contact inhibition and initiating stratified cell growth in well-differentiated human endometrial cancer cells (Figure 1) [4]. While macropinocytosis, which is Rac-dependent, occurs through the temporary split of adjacent cell junctions; there is no protrusion of the cell membrane prior to macropinocytosis. The triggering of this macropinocytosis is based on a transient reduction in epithelial homeostasis by ligand stimulation of the tight junction-associated protein lipolysis-stimulated lipoprotein receptor (LSR).

## 4. Disruption of Epithelial Homeostasis Triggers Cancer Malignancy

### 4.1. Regulation of Epithelial Homeostasis by Cell Polarity

To maintain homeostasis in epithelial cells, it is necessary to establish an adhesion apparatus between adjacent cells and concomitantly establish cell polarity [36]. Adherence junction formation precedes cell–cell junction formation [37]. Adherence junctions interact with the actin cytoskeleton through the nectin-afadin and cadherin-α/β-catenin pathways [38]. The actin cytoskeleton forms circumferential actomyosin bundles and increases the cell thickness [39]. Shroom3 and Lulu1/2 activate circumferential actomyosin bundles via ROCK [40,41]. Tight junctions are established by polar proteins PAR3/PAR6/aPKC, which interact with circumferential actomyosin bundles via ZO1/2/3 [42]. In mature epithelial cell sheets with sufficient epithelial barrier function, occludin (OCLN), claudins (CLDNs), and JAM-A are localized in the tight junction region between adjacent cells, whereas tricellulin (Tric) accumulates in the tricellular contact region in an LSR dependent manner. The LSR is required for the localization of Tric to the tricellular contact region [43], but the mechanism by which LSR accumulates in this region remains unclear. OCLN, CLDNs, and Tric are transmembrane proteins with N- and C-termini in the cytoplasm, whereas JAM-A and LSR are single-transmembrane proteins with one or more immunoglobulin-like domains in the extracellular N-terminal region [44]. The LSR is a receptor for apolipoprotein B (ApoB) and ApoE [45], which are associated with lipid metabolism in hepatocytes [46]. LSR localization is dependent on the cell density in epithelial cells. During the establishment of epithelial homeostasis, LSRs are distributed broadly in the bicellular junction region and gradually accumulate in the tricellular contact region [47]. This change in localization is reversible. LSR withdrawal from the tricellular contact region impairs epithelial homeostasis [48]. Disruption of epithelial barrier function abolishes cell polarity and promotes dedifferentiation [44,49].

### 4.2. Regulation of Epithelial Homeostasis by Cell Density

The cell adhesion apparatus senses cell density [50]. Epithelial cells have a contact inhibition mechanism, which is one of the mechanisms that maintains cell density [51,52]. Contact inhibition involves two mechanisms: growth and motility inhibition [53,54]. These contribute to the regulation of cell number during normal development and wound healing [55]. Physical cell loss and carcinogenesis disrupt contact inhibition [56,57]. Loss of contact inhibition and disruption of cell polarity are typical features of the malignant transformation of cancer cells. These molecular mechanisms involve PAR3, TGF-β, and Hippo pathways [53,58]. KRAS mutations are closely associated with the growth of malignant cells [59]. KRAS mutations are typical oncogenes found in one-quarter of adenocarcinomas [60]. KRAS mutations have been found in endometrial carcinomas and adenomyosis [61,62].

Sawano cell was established by endometrial adenocarcinoma of uterus [63]. Sawano has a KRAS G13D heterozygous mutation and wild-type BRaf [4]. According to this study, Sawano cells have high planar motility at low cell densities, whereas at high cell densities, their growth and motility are temporarily arrested due to contact inhibition. After a certain period of time under high cell density conditions, Sawano initiated multilayered cell growth. In addition, in the presence of MAPK inhibitors, the cells maintained a highly differentiated state. Thus, Sawano cells are a good model for analyzing the malignant transformation process of endometrial carcinoma.

## 5. LSR-Related Molecular Mechanisms Leading to Disruption of Endometrial Homeostasis

### 5.1. Disruption of Endometrial Homeostasis by TGF-β Signaling

TGF-β is associated with increased migration and malignant transformation of endometrial carcinomas through activation of estrogen-related receptor alpha (ERRα); it is also associated with invasion and epithelial-to-mesenchymal transition (EMT) [64]. TGF-β increases HMGB1 expression and induces changes in LSR localization and downregulation, resulting in reduced epithelial barrier homeostasis [65,66,67,68,69,70,71,72]. TGF-β levels are also elevated in high-grade stages of endometriosis [73]. TGF-β is released from endometrial epithelial cells, endometrial stromal cells, endometriotic mesenchymal stem cells, and peritoneal mesothelial cells and regulates endometriosis pathogenesis [74].

### 5.2. Disruption of Endometrial Homeostasis by Hippo-Yap Pathway

The Hippo-YAP pathway is involved in the regulation of cell number and apico-basal polarity. This pathway is involved in tight junctions, adherens junctions, the planar cell polarity (PCP) pathway, the mechanotransduction pathway, and growth factor signaling [52]. YAP also regulates the cell size [75]. YAP is localized in the nucleus, resulting in the activation of transcription factors in various cell types, whereas YAP is degraded in the cytoplasm, resulting in transcriptional repression in smaller and columnar cells [76]. In endometriotic tissue, YAP is localized in the cytoplasm, whereas in G1, G2, and G3 stages of endometrioid carcinomas, YAP is localized in the nucleus [77,78]. LSR knockdown enhanced cell motility and invasion in Sawano cells, and YAP knockdown resulted in cell quiescence [77]. Dobutamine, a β-adrenergic receptor agonist, decreases nuclear YAP levels and increases cytoplasmic phosphorylated YAP levels in human U2OS cells [79]. Dobutamine has also been reported to suppress the high expression of YAP in gastric carcinoma, resulting in suppression of cell motility and invasion [80]. In LSR-knockdown Sawano cells, dobutamine treatment suppressed the enhancement of cell motility and invasion, associated with an increase in the amount of phosphorylated YAP [77]. Activation of the transcription factor TEAD by YAP increased the expression of genes associated with cell proliferation and survival [81]. LSR-knockdown Sawano cells induce expression of the Hippo pathway transcription factor TEAD and inducible gene amphiregulin (AREG) [77]. Immunohistochemical staining analysis using tissues from endometriosis and endometrial cancer revealed that the expression of AREG, an EGFR ligand, is observed in the cytoplasm of cells forming endometriotic tissue and that its expression level increases with increasing cancer stage. In Sawano cells, cell motility and invasion, both of which were increased by LSR knockdown, were significantly suppressed by AREG knockdown. Increased expression of AREG has also been observed in the human pancreatic cancer cell line HPAC, wherein LSR is knocked down, and in poorly differentiated pancreatic ductal adenocarcinomas (PDAC), wherein no LSR is localized in the junctional region [66]. Thus, the downregulation of epithelial barrier function caused by the suppression of LSR expression is involved in the regulation of cell motility and invasion through TEAD-dependent AREG expression via the YAP-Hippo pathway.

## 6. Pathophysiological Changes in Endometriosis and Endometrial Carcinoma Are Accompanied by Changes in LSR Localization

The endometrium is a regenerative tissue that undergoes periodic proliferation and differentiation under the influence of estrogen, progesterone, growth factors, and various cytokines. Menstrual cycle influences the expression and localization of both bicellular and tricellular tight junction proteins. Bicellular tight junction proteins such as CLDN-1, -3, -4, and -7 and OCLN accumulate in the subapical region during the proliferative phase of the endometrium, whereas they are distributed in the basolateral region during the secretory phase [82]. Levels of tricellular tight junction proteins also change dynamically. Both Tric and LSR accumulate in the subapical region during the proliferative phase of the endometrium, wherein Tric is localized in the subapical region during the secretory phase and LSR is widely distributed from the subapical region to the basolateral region [83].

A downregulation of expression of OCLN and CLDN-3, -4, and -7 has been observed in endometriosis [84], and an upregulation of expression of CLDN-3 and -4 has been reported in endometrial cancer [85]. The expression of tricellular tight junction proteins is altered during endometrial pathogenesis [77,83]. These studies demonstrate that the localization of LSR to tricellular contact regions is required for the maintenance of robust endometrial epithelial barrier function. For example, in endometriosis, Tric accumulates in the subapical region, as in the normal human endometrium, whereas LSR is widely distributed not only in the subapical region, but also in the lateral region. In endometrial carcinoma G1, in which most of the gland-like tissue remains, both Tric and LSR are heterogeneously distributed from the subapical to the lateral regions. In stages G2 and G3, the expression levels of both are decreased. Among endometrial cancer-derived cell lines, Sawano, HHUA, and JHMUE-1 cells, which exhibit high to moderate differentiation, express LSR and Tric, whereas spindle-shaped and fibroblast-like JHMUE-2 cells rarely express these markers. Knockdown of LSR in Sawano cells resulted in decreased epithelial barrier function and increased cell motility, invasion, and proliferation, as described above.

In the gland-like structure region of endometrial cancer tissue, upregulation of LSR expression and downregulation of CLDN-1 expression have been observed by immunohistochemical staining analysis [86]. By contrast, in the invasive front area, LSR expression is downregulated and CLDN-1 expression is markedly upregulated. The report has also shown that, CLDN-1 expression upregulates in LSR knocked-down Sawano cells, while the expression levels of OCLN, CLDN-3, -4, and -7 barely change. Prior to LSR knockdown, CLDN-1 is localized in the subapical region, but after the knockdown, it is widely distributed from the subapical region to the basolateral region. Thus, there is a reciprocal relationship between the expression of LSR and CLDN-1, yet almost no regulatory mechanism has been identified. Binding of Sp1 to the CLDN-1 promoter region is required for the regulation of CLDN-1 expression in intestinal epithelium-derived cells [87]. Sp1-binding sites exist in the promoter region of CLDN-1, -4, and -19 [88,89,90]. Sp1 is also involved in the upregulation of CLDN-1 expression in Sawano cells [86]. Sp1 contributes to the expression of matrix metalloproteinases (MMPs) in Sawano cells.

In oral squamous cell carcinoma (OSC) cells expressing CLDN-1, cell invasion is enhanced through cleavage of laminin-5γ2 chains by increased activity of MMP-14 (MT-MMP-1) and MMP-2 [91]. Human colorectal cancer SW480 expressing CLDN-1 have been reported to enhance cell invasion through the activation of MMP-2 and -9 [92]. The initial stage of cell invasion requires reconstitution of the extracellular matrix composition, in addition to the depletion of intercellular junctions [93]. To date, 24 MMPs have been identified [94]. MMP expression varies with the menstrual cycle [95]. MMP-3, -7, -9, and-13 are associated with disease susceptibility in endometriosis [96]. TGF-β is involved in the enhancement of MMP-2 and -3 secretion [97]. LSR knockdown increased the expression levels of MMP-2, -9, -10, and -14 in Sawano cells [86]. MMP-14 is an initiator that activates the MMP cascade by activation of proMMP-2 [98]. In Sawano cells, double knockdown of LSR and CLDN-1 suppresses cell invasion enhanced by LSR knockdown [86]. While some studies have linked increased expression of TGF-β with CLDN-1 expression [99], there exist conflicting reports [100].

## 7. Internalization Pathways of Tight Junction Proteins

Tight junction complexes form static rigid seals, whereas their components exhibit dynamic behavior. The exclusion of tight junction proteins from the cell surface membrane is associated with internalization by clathrin-, caveolae-, and raft-mediated pathways, as well as by macropinocytosis [101]. In intestinal epithelial cell T84, tight junction proteins OCLN, CLDN-1, and JAM-A are internalized via the IFN-γ-induced macropinocytosis-like pathway [102]. In contrast, in T84 cells, *Escherichia coli* cytotoxic necrotizing factor-1 (CNF-1) induces caveolae-dependent intracellular translocation of OCLN without affecting the localization of the AJ proteins E-cadherin and β-catenin [103]. In depolymerized MDCK cells, in which actin filamentation is inhibited by latrunculin A, OCLN is internalized via the caveolae [104]. GFP-CLDN-3 was incorporated from the lateral region by vesicles that incorporated the plasma membrane of two adjacent cells [105]. In Caco-2 cells, Tric, OCLN, CLDN-4, and ZO-1 are excluded from tight junctions in a poly L-arginine-dependent manner. However, the underlying mechanism remains unknown [106]. In bovine retinal endothelial cells (BRECs), OCLN is phosphorylated and ubiquitinated 30 min after VEGF stimulation and translocates from tight junctions to early and late endosomes [107]. LSR is excluded from tricellular tight junctions by LSR ligand stimulation, and LSR subsequently colocalizes with Rab5-positive vesicles at bicellular junctions [4]. The pathway by which the LSR translocates from the bicellular region to the intracellular region and the mechanism by which the intracellularly translocated LSR is recycled to the junction region is unclear.

## 8. Regulation of Cell Motility by Macropinocytosis Induced by LSR Ligand

As mentioned above, decreased expression levels and altered localization of LSR, which accumulates in tricellular tight junctions, alter the localization of Tric, leading to a decrease in the robustness of the epithelial barrier function. Many of these findings were obtained through LSR knockdown experiments, in which it is difficult to temporally control the suppression of gene expression. Hence, Kondoh et al. established an experimental system that enables the simultaneous reduction of LSR expression in all areas of cultured cells using LSR ligands [108]. Administration of the LSR ligand to Sawano cells induces LSR localized in tricellular tight junctions into Rab5-positive intracellular vesicles via bicellular junctions [4]. Under these conditions, the LSR ligand transiently reduced epithelial barrier function, but it had little effect on OCLN expression and localization and did not completely disrupt epithelial barrier function. TEM analysis revealed the presence of tight junctions, adherens junctions, and desmosomes in bicellular junctions. Macropinocytosis does not disrupt the tight junction barrier in a blood-tumor barrier model in rat gliomas [109].

**Figure 2:**
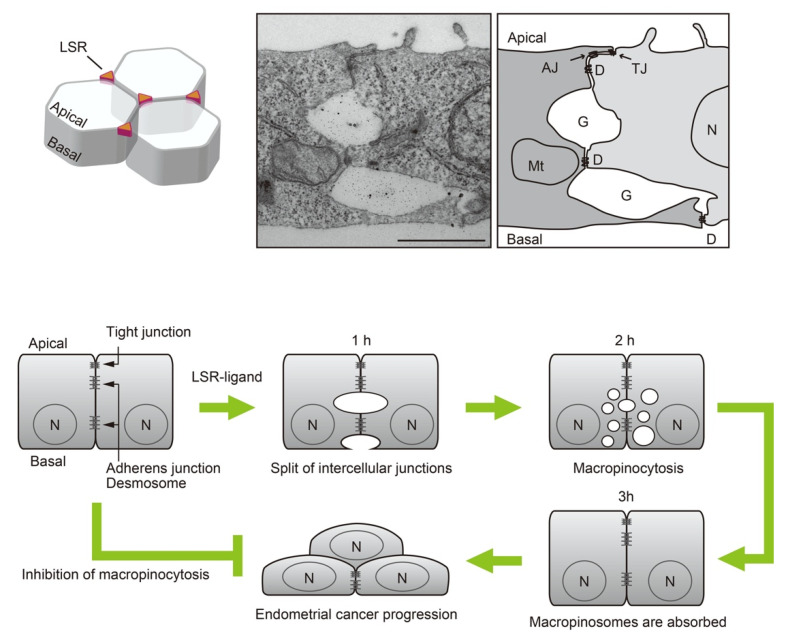
Schematic diagram of the LSR ligand-induced transient macropinocytosis derived from intercellular split. (**Upper panels**) In mature epithelial cells, LSR accumulates in apical tricellular junctions. Administration of the LSR ligand splits the adjacent intercellular spaces, leading to macropinocytosis. The intercellular adhesion apparatus was not disrupted by macropinocytosis. G, intercellular gap; TJ, tight junctions; AJ, adherens junctions; D, desmosomes; N, nucleus; Mt, mitochondria. Bar, 1 μm. (**Lower panels**) The gap between adjacent cells expanded and filled with extracellular fluid, one hour after administration of the LSR ligand. Two hours after addition, numerous macropinosomes that incorporated the extracellular fluid appeared in the cytosol. The vesicles disappeared three hours after addition. The cells then initiated malignant cell growth, resulting in multilayered cell growth. This pathway was suppressed in the presence of a macropinocytosis inhibitor.

The LSR ligand is a partial C-terminal peptide of the *Clostridium perfringens* iota-toxin b subunit. LSR bound to *C. perfringens* iota toxin translocates intracellularly through membrane rafts [110]. LSR expressed in mouse vascular endothelial cells is involved in the establishment of barrier function [111]. Administration of the LSR ligand to mice transiently reduces the endothelial cell barrier function of the blood–brain barrier (BBB) and enhances drug entry into the brain [112]. In the human cancer cell lines Sawano and HPAC, administration of the LSR ligand decreased LSR expression and induced a reduction in barrier function, resulting in enhanced malignant transformation of cancer cells [66,113]. In contact-inhibited monolayers of Sawano cells under high cell density conditions, administration of the LSR ligand resulted in transient and rapid enhancement of planar cell motility [4]. This process requires macropinocytosis, initiated by the cleavage of the gap between adjacent cells (Figure 2). LSR ligand-dependent pinocytosis does not occur in apical or basal membranes. Additionally, no significant accumulation of actin filaments was observed during macropinosome formation. In the presence of the Rac inhibitor NSC23766 or in cells expressing a dominant negative Rac, LSR ligand-dependent macropinocytosis is inhibited. Rac activity is required for JNK activation and is involved in the subsequent enhancement of cell motility. Rac activates JNK [114]. JNK is associated with the regulation of cell motility in various cancer cells [115]. In Sawano cells, LSR ligand stimulation enhances cell motility through JNK activation [113]. JNK inhibitors or knockdown of JNK not only suppresses cell motility but also inhibits macropinocytosis [4]. JNK-mTOR pathway is associated with macropinocytosis in HeLa cells [116].

The mechanism by which cell motility is enhanced following macropinocytosis in DCs has been elucidated. In mature DCs that have undergone macropinocytosis, decreased mTORC1 activity induces the expression of the lysosomal calcium channel TRPML1 and an increase in calcium ions, followed by enhanced cell motility via the activation of myosin II [117]. Myosin II and ROCK play a central role in cell motility associated with actomyosin reorganization [118]. Between neighboring adjacent junctions of Sawano cells, myosin phosphatase MYPT1 accumulates during the contact inhibition phase and phosphorylated myosin accumulates during the cell motility phase [47]. The myosin II inhibitor blebbistatin and the ROCK inhibitor Y27632 did not affect the formation of macropinocytosis, suggesting that LSR ligand-induced macropinocytosis triggers the initiation of planar cell motility.

## 9. SLC9A1-Mediated Macropinocytosis and Its Associated Regulation of Cancer Malignancy

The amiloride derivative EIPA targets SLC9A1 and suppresses macropinocytosis [119]. SLC9A family of proteins are involved in maintaining intracellular pH homeostasis [120]. These proteins were formerly named as sodium hydrogen exchanger (NHE) family proteins [121]. EIPA inhibits the activation of Rac and Cdc42 by acidifying the intracellular pH and inhibiting macropinocytosis by preventing the translocation of Arp3 to the plasma membrane [122]. The Arp2/3 complex is involved not only in actin filament branching and protrusion but also in phagocytic cup formation [6,123]. During RSV infection, extracellular budding and spread of RSV are based on the regulation of the Arp2/3 complex by transcribed viral proteins [124].

SLC9A1 is a ubiquitously expressed plasma membrane protein that directly interacts with ERM proteins [125] and ROCK [126] in its C-terminal region. SLC9A1 is a scaffold protein for Raf and ERK1/2 [127]. In triple-negative breast cancer MDA-MB-231 cells, SLC9A1 activation triggers malignant transformation [128]. SLC9A1 is involved in collective cell migration in head and neck squamous cell carcinoma [129]. In A431 cells, an increase in intracellular pH due to SLC9A1 activation is required for EGF-dependent macropinocytosis [122]. SLC9A1 has attracted attention as a novel anticancer drug target [130].

**Figure 3:**
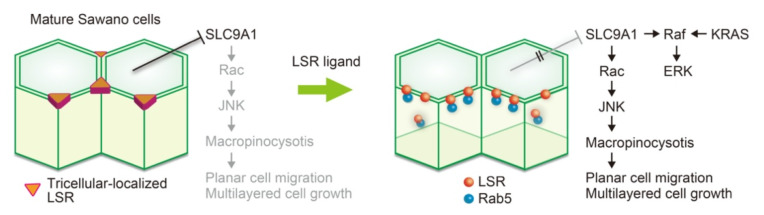
Schematic representation of the mechanism by which endometrial cancer Sawano initiates malignant cell growth. Mature epithelial sheets, where LSR localize at tricellular tight junctions, suppress macropinocytosis. LSR ligand administration leads to the translocation of LSR from tricellular tight junctions. LSR interacts with Rab5 in the bicellular region, resulting in decreased LSR expression. SLC9A1 is presumably activated through a signal that accompanies the alteration of LSR localization, followed by Rac activation. SLC9A1 scaffolds Raf and ERK downstream of KRAS. Activated Rac induces JNK activation, resulting in the formation of macropinocytosis, followed by an increase in planar cell migration, and eventually, multilayered cell growth.

In the presence of EIPA, similar to the knockdown of SLC9A1, few macropinosomes were formed upon treatment with the LSR ligand; furthermore, no cell motility was confirmed under these conditions (Figure 3) [4]. Cryo-EM analysis showed that *C. perfringens* iota toxin, the origin of the LSR ligand, enters the cell under acidic conditions [131]. Transient acidification of the extracellular region by SLC9A1 activation may affect the infection [132]. If the *C. perfringens* infection pathway is similar to the binding of the LSR ligand to the LSR, it is possible that the infection may be involved in the pathway by which genetically mutated KRAS cells awaken from dormancy. KRAS mutations are representative gene mutations in endometrial cancer [133]. As in endometriosis, lesions of adenomyosis and their surrounding normal tissues have KRAS mutations, even though they are not cancerous [62]. LSR-targeted bacterial infections and associated macropinocytosis may be involved in the malignant transformation of these non-cancerous cells. It has been reported that bicellular tight junction proteins, such as OCLN, CLDNs, and JAM-A, are receptors for adenovirus, papillomavirus, rotavirus, influenza virus, severe acute respiratory syndrome (SARS), and HIV-1 [134]. Understanding the mechanisms by which these exogenous factors are incorporated into cells may help avoid infection and carcinogenesis.

Ras mutations, other than the KRAS G13D mutation, are also associated with macropinocytosis. Analysis of human pancreatic adenocarcinoma-derived MIA PaCa-2 cells with KRAS G12C mutation, human bladder carcinoma-derived T24 cells with HRas G12V, and exogenous KRAS G12V-expressing NIH3T3 cells revealed that these cells obtain glutamine needed for proliferation by uptake of albumin from the extracellular fluid via macropinocytosis [135]. In knock-in mice transfected with KRAS G12D into the pancreas, amino acids necessary for cancer cell growth are acquired through macropinocytosis, a mechanism that is rarely observed in normal cells [136]. These findings implicate macropinocytosis between epithelial barrier homeostasis and initiation of malignant transformation in KRAS-mutant cells. Therefore, it is suggested that the epithelial barrier function may negatively regulate the signaling pathways from mutant KRAS in the dormant state Sawano cells, in which LSR is localized in tricellular tight junctions. It is speculated that LSR may be involved in the initiation of the malignant transformation of endometriosis and endometrial cancer.

## 10. Conclusions

Atypical macropinocytosis, which is derived from the partial cleavage between adjacent cells, is essential for the initiation of malignant progression of endometrial cancer cells. Aberrant cell proliferation, disruption of differentiation, and EMT are induced by the activation of oncogenes, including Ras, and the inactivation of tumor suppressor genes, including PTEN and p53. These processes involve reorganization of intercellular junctions. LSR may be an initiating factor in the disruption of epithelial barrier homeostasis. Accumulation of LSR in tricellular tight junctions is necessary to maintain the robustness of epithelial barrier function. As shown in Figure 3, changes in the localization of LSR in tricellular tight junctions induce atypical macropinocytosis. Furthermore, macropinocytosis triggers a rapid increase in the motility of endometrial cancer cells and induction of multilayered cell growth. However, it is unclear when and where macropinocytosis occurs in proliferating cancer cells. It is still unclear whether endogenous LSR ligands are involved in the pathological process of endometriosis and the malignant progression of endometrial cancer. The elucidation of these mechanisms may contribute to the development of new therapeutic methods for these diseases. It is expected that mechanisms underlying the formation of atypical macropinocytosis will be discovered.
